# Kaliumsubstitution – ist der Ersatz von natrium- durch kaliumhaltige Speisesalze sinnvoll?

**DOI:** 10.1007/s00108-026-02112-6

**Published:** 2026-05-05

**Authors:** Felix Götzinger, Michael Kunz, Lucas Lauder, Felix Mahfoud

**Affiliations:** Klinik für Kardiologie, Universitäres Herzzentrum und Cardiovascular Research Institute Basel, Universitätsspital Basel, Universität Basel, Petersgraben 4, 4031 Basel, Schweiz

**Keywords:** Natriumaufnahme, Kaliumaufnahme, Hypertonie, Herzinsuffizienz, Kardiovaskuläre Ereignisse, Sodium intake, Potassium intake, Hypertension, Heart failure, Cardiovascular events

## Abstract

**Hintergrund:**

Bluthochdruck ist die häufigste Ursache für vorzeitige Mortalität weltweit, während Dekompensationen bei Herzinsuffizienz zu den häufigsten Gründen für Krankenhausaufnahmen zählen. Beide Erkrankungen werden durch eine hohe Natriumaufnahme über Speisesalz und verarbeitete Lebensmittel begünstigt. Kalium wirkt hingegen blutdrucksenkend und könnte daher als Ersatz für Natrium in Speisesalzen verwendet werden, um dessen negativen Einfluss auf Hypertonie und Herzinsuffizienz zu reduzieren.

**Ziel der Übersicht:**

In der vorliegenden Übersicht soll die aktuelle Evidenz zu den kardiovaskulären Effekten von natrium- und kaliumhaltigen Speisesalzen zusammengefasst werden.

**Datenlage:**

Epidemiologische Studien zeigen konsistent, dass eine hohe Natriumaufnahme mit erhöhtem Blutdruck und einer höheren Rate kardiovaskulärer Ereignisse assoziiert ist. Metaanalysen belegen zudem, dass eine Reduktion des Natriumkonsums zu einer Abnahme kardiovaskulärer Ereignisse beiträgt. In einer clusterrandomisierten Studie führte der teilweise Ersatz von natrium- durch kaliumhaltige Speisesalze bei arterieller Hypertonie zu einer Reduktion kardiovaskulärer Ereignisse und kardiovaskulärer Sterblichkeit. Robuste Daten zur Reduktion harter klinischer Endpunkte durch Natriumreduktion bei Herzinsuffizienz gibt es gegenwärtig nicht.

**Schlussfolgerung:**

Eine hohe Natriumaufnahme hat nachweislich negative Effekte auf die kardiovaskuläre Gesundheit. Der Ersatz von natrium- durch kaliumhaltige Speisesalze stellt daher einen vielversprechenden Ansatz dar, um die hohe Morbidität und Mortalität von Bluthochdruck zu mindern.

## Abstract (en)

**Background:**

Hypertension is the leading cause of premature death globally, and acute decompensated heart failure is among the most common causes of hospitalization. Both conditions are adversely affected by high dietary sodium intake. In contrast, potassium has been shown to lower blood pressure. Substituting dietary sodium with potassium may therefore reduce morbidity and mortality associated with hypertension and possibly heart failure.

**Objectives:**

This review summarizes the current evidence on the cardiovascular effects of dietary sodium and potassium-containing salts.

**Current data:**

Epidemiological studies consistently demonstrate that high sodium intake is associated with elevated blood pressure and increased rates of cardiovascular morbidity and mortality. Meta-analyses indicate that reducing sodium intake lowers blood pressure and reduces cardiovascular events. In a cluster-randomized trial, partial substitution of sodium chloride with potassium-enriched salt reduced cardiovascular morbidity and mortality in patients with hypertension. However, robust data on the effects of sodium restriction or potassium substitution on hard cardiovascular outcomes in patients with heart failure remain limited.

**Conclusions:**

High sodium intake has detrimental effects on cardiovascular health. Substituting sodium with potassium-containing salts represents a promising strategy to reduce morbidity and mortality associated with hypertension.

Kochsalz (Natriumchlorid [NaCl]) ist ein günstiger Lebensmittelbestandteil, der unter anderem zur Haltbarmachung und Geschmacksverstärkung vieler verarbeiteter Lebensmittel und von „Convenience-Food“ verwendet wird. Schätzungen gehen davon aus, dass jeder Mensch weltweit im Durchschnitt etwa 10 g Kochsalz pro Tag zu sich nimmt [35]. Hinzu kommt, dass viele Produkte verstecktes Salz enthalten, beispielsweise häufig genutzte Brausetabletten [22].

Das im Kochsalz enthaltene Natrium ist insbesondere bei kochsalzsensitiven Menschen mit einem Anstieg des Blutdrucks assoziiert, vornehmlich bei Patienten mit arterieller Hypertonie; bei Patienten mit Herzinsuffizienz kann es zur Flüssigkeits- und Volumenretention beitragen und somit zu einer kardialen Dekompensation führen [18]. Aus diesen Gründen empfiehlt die Weltgesundheitsorganisation, die tägliche Kochsalzaufnahme auf 5 g zu beschränken, was einer Natriummenge von 2,2 g entspricht. Gleichzeitig liegt zunehmend Evidenz vor, die den Einsatz von Kaliumsalzen als Ersatz für Kochsalz unterstützt. Im Gegensatz zu Natriumsalzen senken Kaliumsalze den Blutdruck und gemäß einer kürzlich veröffentlichten Studie führt der Teilersatz von Kochsalz (NaCl) durch Kaliumsalze sogar zu einer Reduktion der kardiovaskulären Sterblichkeit bei Bluthochdruck. Vor diesem Hintergrund fasst der vorliegende Beitrag die aktuelle Evidenz zu den kardiovaskulären Aspekten von Natrium- und Kaliumsalzen zusammen.

## Gesundheitliche Folgen einer hohen Natriumaufnahme

**Abb. 1:**
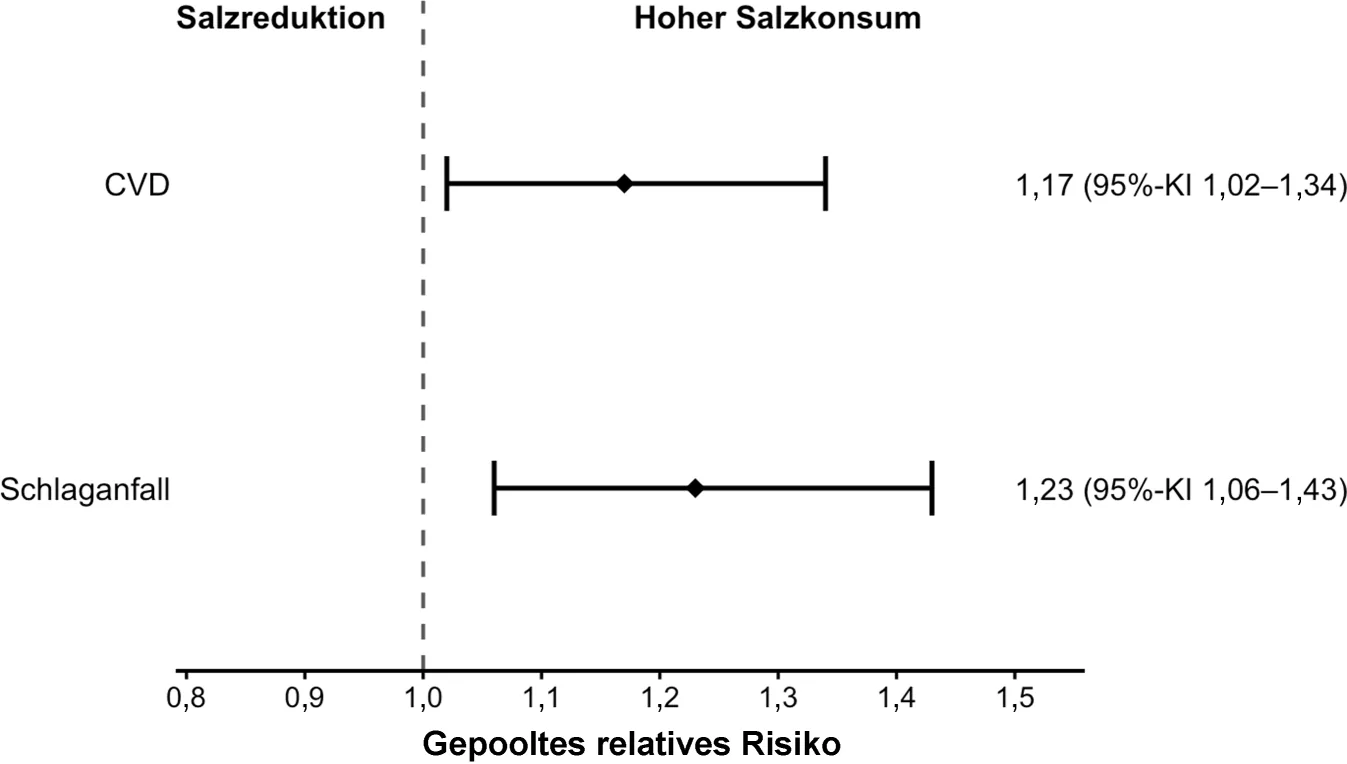
Gepooltes relatives Risiko für Schlaganfälle und kardiovaskuläre Erkrankungen bei hohem Salzkonsum (> 6 g/Tag). *CVD* „cardiovascular diseases“ (kardiovaskuläre Erkrankungen), *KI* Konfidenzintervall. (Adaptiert von Strazzullo et al. [39])

Bluthochdruck ist weiterhin die führende Ursache für ein vorzeitiges Versterben [24]. Dabei gilt ein hoher Salzkonsum von mehr als 5 g/Tag als wichtiger modifizierbarer Risikofaktor für die Entstehung einer arteriellen Hypertonie [35]. Das im Salz enthaltene Natrium erhöht den Vasotonus und führt zur Flüssigkeitsretention sowie zum Remodeling der Blutgefäße [40]. Präklinische und klinische Studien zeigen, dass die individuelle Verträglichkeit von Natrium (Salzsensitivität) unterschiedlich ist und dass nicht alle Individuen auf Natriumkonsum mit einem Blutdruckanstieg reagieren [30, 33]. Epidemiologische Studien zeigen, dass ein zu hoher Salzkonsum, vermittelt über Bluthochdruck und damit assoziierte Folgeerkrankungen, für 1,9 Mio. Todesfälle und 44 Mio. „disability-adjusted life years“ weltweit und pro Jahr verantwortlich sein könnte [2, 6]. Metaanalysen zeigen eine erhöhte Rate an kardiovaskulären Erkrankungen bei hohem Salzkonsum (> 6 g/Tag; Abb. 1; [39]).

Basierend auf einer Analyse der UK Biobank könnte außerdem das Nachsalzen von Speisen mit einer erhöhten Mortalität assoziiert sein. Das Risiko für die Gesamtsterblichkeit war bei Menschen, die ihre Speisen immer nachsalzten, im Vergleich zu denen, die nie oder nur selten das Essen nachsalzten, um 28 % erhöht (Hazard Ratio [HR] 1,28; 95 %-Konfidenzintervall [KI] 1,20–1,35; *p* < 0,001; [23]). Die Plausibilität dieses Zusammenhangs wurde zusätzlich dadurch gestützt, dass die Natriumausscheidung im Urin mit zunehmendem Nachsalzen höher war [23].

**Abb. 2:**
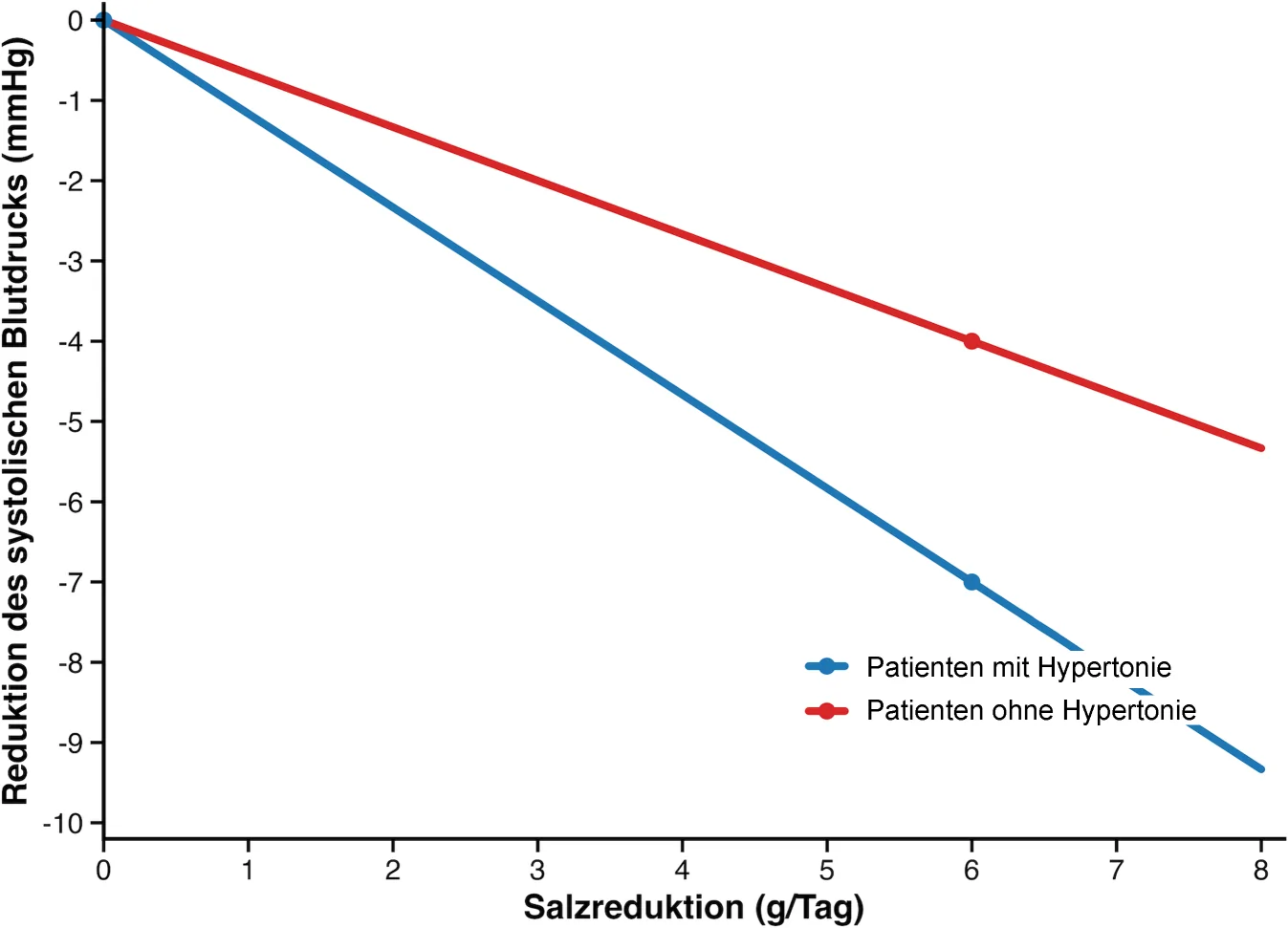
Mögliches lineares Verhältnis zwischen Salzreduktion und Blutdruckreduktion. (Adaptiert von He et al. [16])

Gleichzeitig zeigen Metaanalysen, dass eine Reduktion des Natriumkonsums mit einer geringeren Inzidenz kardiovaskulärer Ereignisse assoziiert ist [19]. Dies wird wahrscheinlich über blutdrucksenkende Effekte der Natriumrestriktion vermittelt. Es wird postuliert, dass jede Reduktion der Natriumexkretion um 50 mmol mit einer Senkung des systolischen Blutdrucks um 1,10 mm Hg einhergeht [19]. Gemäß einem Cochrane-Review ist eine Reduktion der Natriumaufnahme um 1,8 g/Tag (entspricht 4,6 g Kochsalz am Tag) mit einer Reduktion des systolischen und diastolischen Blutdrucks um 5,1 bzw. 2,7 mm Hg assoziiert [17]. Auch andere Studien zeigen, dass wahrscheinlich ein nahezu lineares Verhältnis zwischen Salzreduktion und Reduktion des Blutdrucks besteht (Abb. 2; [16]). Es gibt allerdings auch Theorien, die eine u‑förmige oder eine gesättigte Verbindung zwischen Natrium und der Blutdruckhöhe sowie den nachfolgenden kardiovaskulären Ereignissen annehmen. Diese unterschiedlichen Zusammenhänge werden durch unterschiedliche Untersuchungsmethoden hinsichtlich des Natriumkonsums erklärt, die alle unterschiedlich sensitiv sind [16–18].

Die Weltgesundheitsorganisation und die Leitlinien der European Society of Cardiology (ESC) empfehlen eine Reduktion des Natriumkonsums auf maximal 2 g/Tag für alle Patienten mit erhöhtem Blutdruck (≥ 130/70 mm Hg) oder arterieller Hypertonie (≥ 140/90 mm Hg; [25, 27]). Die Nationale Versorgungs Leitlinie Hypertonie empfiehlt, bei Bluthochdruck nicht mehr als 2,4 g Natrium (etwa 6 g Kochsalz) täglich zu konsumieren ([5]; Tab. 1).

**Tab. 1:** Aktuelle Empfehlungen zum Natrium- und Kaliumkonsum der Fachgesellschaften

Fachgesellschaft	Natrium	Kalium
*Arterielle Hypertonie*		
AHA 2025	≤ 2,3 g/Tag (entspricht ca. 6 g/Tag Kochsalz)	3,5–5 g/Tag
ESC 2024	≤ 2 g/Tag (entspricht ca. ≤ 5 g/Tag Kochsalz)	≥ 3,5 g/Tag
ESH 2023	≤ 2 g/Tag (entspricht ca. ≤ 5 g/Tag Kochsalz)	≥ 3,5 g/Tag
NVL 2023	≤ 2,4 g/Tag (entspricht ca. 6 g/Tag Kochsalz)	Keine Empfehlung
*Herzinsuffizienz*		
ESC HFA 2024	1,5–4,0 g/Tag (entspricht ca. 4–7 g/Tag Kochsalz)	Keine Empfehlung
AHA 2022	≤ 2,3 g/Tag (entspricht ca. 6 g/Tag Kochsalz)*	Keine Empfehlung
NVL 2023	Keine Empfehlung	Keine Empfehlung

*AHA* American Heart Association, *ESC* European Society of Cardiology, *ESC HFA* Heart Failure Association of the European Society of Cardiology, *ESH* European Society of Hypertension, *NVL* Nationale Versorgungsleitlinie

*Schwache Empfehlung („limited data“)

In der klinischen Praxis wird eine Salzreduktion nicht nur bei arterieller Hypertonie empfohlen, sondern häufig auch bei Herzinsuffizienz [26]. Ein hoher Salzkonsum kann zu einer vermehrten Volumenretention beitragen und so eine kardiale Dekompensation begünstigen. Indirekte Hinweise hierauf lieferte eine Studie, in der Patienten mit Herzinsuffizienz untersucht wurden, die natriumhaltige Brausetabletten, beispielsweise lösliches Paracetamol, einnahmen. In der Studie zeigte sich, dass der Gebrauch solcher Brausetabletten mit einem erhöhten Risiko für Hospitalisationen einherging (adjustierte Odds Ratio [aOR] 1,56; 95 %-KI 1,27–1,90; *p* < 0,001; [34]). Eine weitere Studie ergab, dass das Risiko für kardiovaskuläre Ereignisse bei Patienten, die regelmäßig natriumhaltige Brausetabletten einnahmen, um 16 % (aOR 1,16; 95 %-KI 1,12–1,21) höher war als bei denen, die nicht regelmäßig Brausetabletten zu sich nahmen [15].

Hoher Salzkonsum kann die Volumenretention verstärken und eine kardiale Dekompensation begünstigen

Im Gegensatz zur arteriellen Hypertonie konnte bisher allerdings nicht konsistent belegt werden, dass eine Salz- und Natriumrestriktion bei Herzinsuffizienz zu einer Reduktion harter Endpunkte führt. Allerdings zeigen Studien eine Verbesserung der Lebensqualität [11]. Die SODIUM-HF-Studie randomisierte beispielsweise 800 Patienten mit einer Herzinsuffizienz (sowohl mit erhaltener als auch mit reduzierter Pumpfunktion) in den New-York-Heart-Association(NYHA)-Stadien II oder III zu einer reduzierten Natriumaufnahme von < 1,5 g/Tag oder zum Behandlungsstandard [14]. Alle Patienten erhielten dabei die bestmögliche medikamentöse Herzinsuffizienztherapie. Der primäre Endpunkt war ein kombinierter Endpunkt aus kardiovaskulär bedingter Krankenhausaufnahme, kardiovaskulär bedingtem Besuch der Notaufnahme sowie Gesamtmortalität innerhalb von 12 Monaten. In der Studie führte die Natriumreduktion nicht zu einer Reduktion dieses kombinierten Endpunkts (HR 0,89; 95 %-KI 0,63–1,26; *p* = 0,53). Zusätzlich wurden keine der Einzelkomponenten signifikant reduziert [14]. Interessanterweise lag der mediane Natriumkonsum in der Interventionsgruppe während der Studie bei etwa 1,7 g/Tag und damit über dem gesetzten Ziel von 1,5 g/Tag, was die Herausforderungen der Umsetzbarkeit einer derartigen Natriumreduktion widerspiegelt. Die Studie zeigte eine leichte Verbesserung der Lebensqualität gemessen mit dem Kansas City Cardiomyopathy Questionnaire (Differenz von 3,4 Punkten zwischen den Gruppen zugunsten der Natriumrestriktion; *p* = 0,011) sowie der NYHA-Klasse [14]. Diese subjektiven Endpunkte müssen infolge des offenen Studiendesigns aber kritisch beurteilt werden [3]. Infolge dieser und weiterer Studien sind die Empfehlungen zur Natriumreduktion in aktuellen Richtlinien zur Herzinsuffizienz weniger klar formuliert. So empfiehlt ein Positionspapier der Heart Failure Association of the ESC eine Natriumreduktion auf 1,5–4,0 g/Tag für die meisten Patienten (Tab. 1; [29]). Die Leitlinien der American Heart Association (AHA) geben eine ähnliche Empfehlung und weisen auf die limitierte Evidenz hin.

Neben Effekten auf das kardiovaskuläre System ist ein hoher Natriumkonsum auch mit anderen Erkrankungen assoziiert [18]. Beispielsweise trägt er zur Entstehung und Progression einer chronischen Nierenerkrankung bei [37]. Zusätzlich führt ein hoher Plasmanatriumspiegel zu einer gesteigerten renalen Kalziumausscheidung, was zur Entstehung von Nierensteinen und Osteoporose beitragen kann [7, 13]. Außerdem ist ein hoher Natriumkonsum mit der Entstehung von Magenkarzinomen assoziiert [12].

Ein hoher Natriumkonsum trägt zur Entstehung und Progression einer chronischen Nierenerkrankung bei

Studien, die neutrale oder gar positive Effekte einer Natriumeinnahme nachgewiesen haben, gibt es hingegen nur wenige. Eine große ökologische Studie erfasste den Natriumgebrauch von Menschen in mehr als 180 Ländern und ermittelte mittels logistischer Regression die Zusammenhänge zwischen dem Natriumgebrauch und der Sterblichkeit jeglicher Ursache. Die Studie fand dabei eine Abnahme der Sterblichkeit bei zunehmendem Natriumkonsum bis 6 g/Tag [28]. Mutmaßlich sind diese Effekte durch das Studiendesign und den epidemiologischen Charakter der Studie erklärbar, und durch die damit einhergehende inhärente Limitation, dass für viele Einflussfaktoren nicht korrigiert werden kann. Randomisierte Studien, die eine Reduktion der Sterblichkeit durch Natriumsteigerung zeigen, gibt es bisher nicht.

## Kann Kalium helfen?

**Abb. 3:**
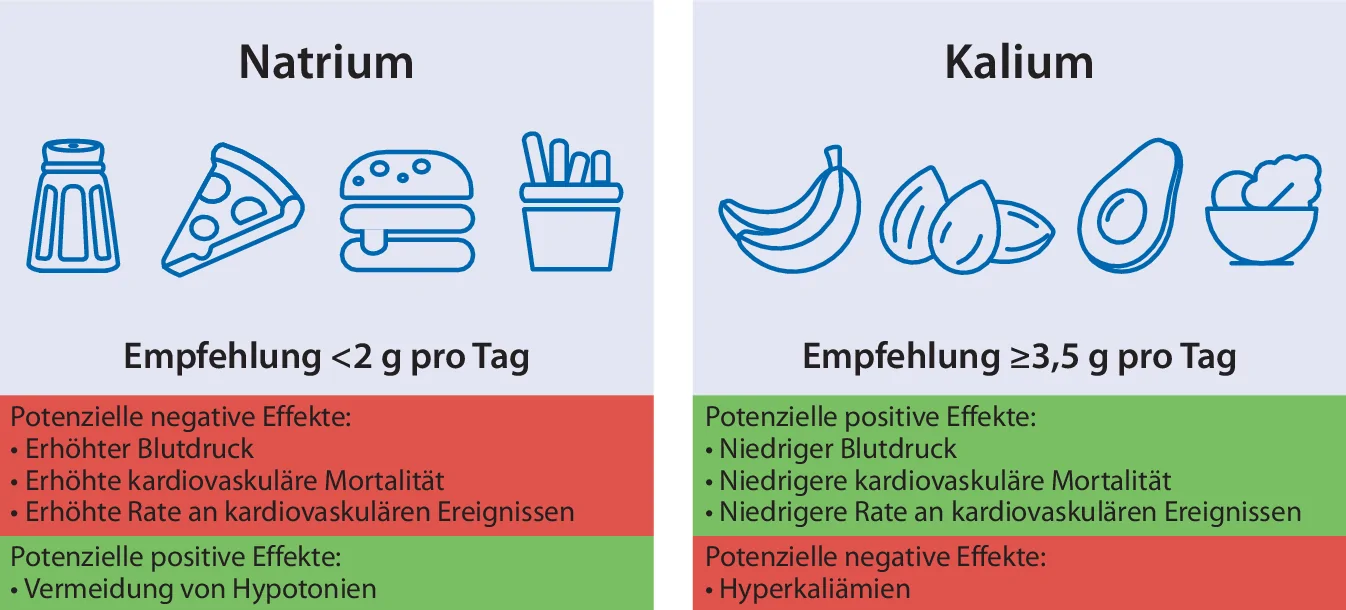
Natrium- und kaliumhaltige Lebensmittel sowie aktuelle Empfehlungen der Weltgesundheitsorganisation zur Aufnahme von Natrium und Kalium mit der Nahrung. Potenzielle positive und negative Effekte

Kalium kann blutdrucksenkende Effekte aufweisen [1]. Daher wird von Fachgesellschaften eine hohe Kaliumaufnahme empfohlen (3,5–5 g/Tag; [9, 25, 27]). Kalium ist unter anderem in Früchten und Gemüse, Hülsenfrüchten, Nüssen und Milchprodukten enthalten (Abb. 3). Als besonders kaliumhaltig gelten Avocados, Bananen und Spinat [27]. Allerdings werden die von Fachgesellschaften empfohlenen Zielwerte in vielen Ländern nicht erreicht und der durchschnittliche globale Konsum von Kalium liegt nur bei etwa 2,25 g/Tag [36].

Aus diesen Gründen wurden Studien durchgeführt, die einen pragmatischen Ersatz von natriumhaltigen Speisesalzen durch kaliumhaltige Speisesalze untersuchten (Tab. 2; [4, 8, 42]). Der Ersatz von Natrium durch Kalium trägt dazu bei, dass der Natriumkonsum reduziert und gleichzeitig der Kaliumkonsum erhöht wird. Beides sollte positive gesundheitliche Konsequenzen haben. Die randomisierte SSaSS-Studie untersuchte bei knapp 21.000 Patienten aus 600 Dörfern in China den normalen Gebrauch von Speisesalz im Vergleich zu einem kaliumhaltigen Ersatzsalz (25 % Kaliumchlorid, 75 % Natriumchlorid; [31]). Eingeschlossen wurden Patienten mit einem Schlaganfall in der medizinischen Vorgeschichte oder Patienten, die älter als 60 Jahre waren und eine arterielle Hypertonie aufwiesen. Die Patienten wurden für 4,7 Jahre nachbeobachtet. Wie dabei gezeigt werden konnte, führte der Teilersatz des Speisesalzes durch Kaliumsalz zu einer signifikanten Reduktion von Schlaganfällen (29 vs. 34 Ereignisse pro 1000 Personenjahre; Rate Ratio 0,86; *p* = 0,006), kardiovaskulären Ereignissen (49 vs. 56 Ereignisse pro 1000 Personenjahre; Rate Ratio 0,87; *p* < 0,001) und Todesfällen (39 vs. 45 Ereignisse pro 1000 Personenjahre; Rate Ratio 0,88; *p* < 0,001). Gleichzeitig war die Kaliumsubstitution mit einem guten Sicherheitsprofil assoziiert [31]. Bei der Interpretation der Ergebnisse ist der Hinweis wichtig, dass die Studie in China durchgeführt wurde. Zum einen ist eine Generalisierbarkeit auf andere Ethnien damit nicht vollständig möglich. Zum anderen bestand die Diät der Studienteilnehmer vorwiegend aus nichtprozessierten Lebensmitteln und der Ersatz von 25 % des Speisesalzes hatte damit einen hohen relativen Anteil an der Gesamternährung bzw. der Gesamtnatriumaufnahme [20]. In der westlichen Welt ist der Anteil an verarbeiteten Lebensmitteln in der Ernährung hingegen höher. Ob der Teilersatz des Speisesalzes also in Populationen mit anderen Essgewohnheiten zu gleichen Effekten führt, ist unklar [20]. Basierend auf den oben geschilderten Daten empfehlen die aktuellen Leitlinien der European Society of Hypertension (ESH), ESC und AHA die Substitution natriumhaltiger mit kaliumhaltigen Speisesalzen für Patient mit arterieller Hypertonie [41].

**Tab. 2:** Zusammenfassung großer Studien (*n* > 500) zur Substitution natriumhaltiger mit kaliumhaltigem Speisesalz

Name der Studie	Autor, Jahr	Population	Intervention	Outcomes	Sicherheit
Taiwan Veterans Study	Chang et al. [8]	1981 Veteranen in Taiwan mit und ohne Hypertonie	Vollständiger Ersatz des normalen Speisesalzes mit einem 49 %-kaliumhaltigen Speisesalz	Reduktion der kardiovaskulären Sterblichkeit um 41 % (aHR 0,59; 95 %-KI 0,37–0,95)	Nicht berichtet
Peru Salt Substitute Trial	Bernabe-Ortiz et al. [4]	2376 Bürger in Peru mit und ohne Hypertonie	Vollständiger Ersatz des normalen Speisesalzes mit einem 25 %-kaliumhaltigen Speisesalz	Reduktion des Auftretens einer Hypertonie um 51 % (HR 0,49; 95 %-KI 0,34–0,71)	Keine negativen Effekte
DECIDE-Salt Study	Yuan et al. [42]	1612 Bewohner von Altersheimen in China mit und ohne Hypertonie	Vollständiger Ersatz des normalen Speisesalzes mit einem 25 %-kaliumhaltigen Speisesalz	Reduktion des systolischen Blutdrucks (−7 mm Hg; 95 %-KI −10 bis −4 mm Hg), Reduktion des Auftretens kardiovaskulärer Ereignisse (HR 0,60; 95 %-KI 0,38–0,96)	Höhere Rate an biochemischen Hyperkaliämien (RR 3,3; 95 %-KI 1,5–7,5) ohne klinische Konsequenzen
SSaSS Study	Neal et al. [31]	20.995 Bewohner chinesischer Dörfer mit Hypertonie und Alter > 60 Jahre oder mit vorbestehendem Schlaganfall	Vollständiger Ersatz des normalen Speisesalzes mit einem 25 %-kaliumhaltigen Speisesalz	Reduktion von Schlaganfällen (RR 0,86; 95 %-KI 0,77–0,96), kardiovaskulären Ereignissen (RR 0,87; 95 %-KI 0,80–0,94) und Todesfällen (RR 0,88; 95 %-KI 0,82–0,95)	Kein Unterschied im Auftreten unerwünschter Ereignisse

*aHR* adjustierte Hazard Ratio, *HR* Hazard Ratio, *KI* Konfidenzintervall, *RR* Rate Ratio

Leitlinien empfehlen die Substitution mit kaliumhaltigen Speisesalzen bei arterieller Hypertonie

Studien, die den Ersatz von natrium- durch kaliumhaltige Speisesalze bei Patienten mit Herzinsuffizienz getestet haben, sind derzeit nicht verfügbar. Es ist allerdings bekannt, dass es eine U‑Kurven-förmige Assoziation zwischen dem Serumkaliumspiegel und dem Überleben bei Herzinsuffizienz gibt, da sowohl Hypo- als auch Hyperkaliämien mit Arrhythmierisiken assoziiert sind [32]. Als ein idealer Kaliumbereich haben sich dabei 4,0–5,0 mmol/l herausgestellt [21, 32]. Dies zeigte auch eine aktuelle randomisierte Studie, in der Patienten, die bereits einen implantierten Defibrillator hatten, zu einem hohen Serumkalium (4,5–5,0 mmol/l) oder dem Behandlungsstandard randomisiert wurden. Die höheren Kaliumspiegel wurden mit kaliumreicher Ernährung, Kaliumsupplementation und der Verwendung von Mineralokortikoidrezeptorantagonisten erreicht. Die Studie randomisierte 1200 Patienten 1:1 und fand, dass höhere Kaliumspiegel mit einer reduzierten Auftretenswahrscheinlichkeit des primären Endpunkts, bestehend aus Gesamtmortalität, Krankenhausaufnahmen, einer anhaltenden ventrikulären Tachykardie und einer adäquaten Therapie durch den Defibrillator, assoziiert waren (HR 0,76; 95 %-KI 0,61–0,95; [21]). Es traten keine vermehrten Raten von Hyperkaliämien in der Interventionsgruppe auf, und insgesamt war die Kaliumerhöhung sicher. Limitiert wird die Studie durch das offene Design, die hohe Rate an eingeschlossenen Patienten, die zuvor bereits ventrikuläre Tachykardien hatten, und dadurch, dass trotz Infrastruktur der randomisierten Studie etwa ein Drittel der Patienten die Studienmedikamente nicht bis zum Ende einnehmen konnte. Die Generalisierbarkeit der Studie ist also unklar. Die Studie zeigt die Schwierigkeit einer anhaltenden Supplementation von Kalium [10].

Es besteht ein besonderes Risiko für Hyperkaliämien bei gleichzeitiger chronischer Nierenerkrankung. Bei Patienten mit chronischer Nierenerkrankung in den Stadien G3–5, also mit einer glomerulären Filtrationsrate ≤ 60 ml/min pro 1,73 m^2^, sollte nur mit Vorsicht eine Kaliumsupplementation erfolgen, da ein höheres Risiko für Hyperkaliämien besteht [38]. Dies ist von besonderer Bedeutung, da die chronische Nierenerkrankung eine der häufigsten Komorbiditäten kardiovaskulär erkrankter Patienten ist. Die Ergebnisse von SSaSS und anderen Studien weisen allerdings insgesamt darauf hin, dass die Gefahr klinisch relevanter Hyperkaliämien unter Kaliumersatz gering ist (Tab. 2; [4, 8, 31, 42]). Studien zum Einsatz von Kaliumsalzen bei chronischer Nierenerkrankung liegen bislang nicht vor.

## Fazit für die Praxis

- Patienten mit arterieller Hypertonie wird zur Salz- und Natriumrestriktion auf 5 g/Tag bzw. 2 g/Tag geraten.
- Patienten mit arterieller Hypertonie wird dazu geraten, den Kaliumkonsum auf ≥ 3,5 g/Tag zu erhöhen.
- Randomisierte, kontrollierte Studien zeigen, dass der Ersatz von natrium- durch kaliumhaltige Speisesalze zu einer relevanten Reduktion kardiovaskulärer Ereignisse und der kardiovaskulären Mortalität beitragen kann.
- Die Generalisierbarkeit dieser Studien für westliche Ernährungsweisen und die Übertragbarkeit auf nichtasiatische Ethnien sind bisher aber noch unklar.

## Data Availability

Es handelt sich um ein Review, für das nur öffentlich verfügbare Publikationen genutzt wurden. Die Daten können aber bei entsprechender sinngemäßer Anfrage vom korrespondierenden Autor erfragt werden.

## Abkürzungen

AHA: American Heart Association
aOR: Adjustierte Odds Ratio
CVD: „Cardiovascular diseases“ (kardiovaskuläre Erkrankungen)
ESC: European Society of Cardiology
ESH: European Society of Hypertension
HR: Hazard Ratio
KI: Konfidenzintervall
NaCl: Natriumchlorid
NVL: Nationale VersorgungsLeitlinie
NYHA: New York Heart Association
